# Chronic high-sodium diet intake after weaning lead to neurogenic hypertension in adult Wistar rats

**DOI:** 10.1038/s41598-017-05984-9

**Published:** 2017-07-18

**Authors:** Paula Magalhães Gomes, Renato Willian Martins Sá, Giovana Lopes Aguiar, Milede Hanner Saraiva Paes, Andréia Carvalho Alzamora, Wanderson Geraldo Lima, Lisandra Brandino de Oliveira, Sean David Stocker, Vagner Roberto Antunes, Leonardo M. Cardoso

**Affiliations:** 1 Department of Biological Sciences, Institute of Exact and Biological Sciences and NUPEB, Federal University of Ouro Preto, Ouro Preto, (MG) Brazil; 2 Department of Physiology and Biophysics, Institute of Biomedical Sciences, University of Sao Paulo, Sao Paulo, (SP) Brazil; 3 Department of Medicine, Division of Renal-Electrolyte, University of Pittsburgh School of Medicine, Pittsburgh, (PA) United States of America

## Abstract

In this study, we investigated some mechanisms involved in sodium-dependent hypertension of rats exposed to chronic salt (NaCl) intake from weaning until adult age. Weaned male Wistar rats were placed under high (0.90% w/w, HS) or regular (0.27% w/w, Cont) sodium diets for 12 weeks. Water consumption, urine output and sodium excretion were higher in HS rats compared to control. Blood pressure (BP) was directly measured by the arterial catheter and found 13.8% higher in HS vs Cont rats. Ganglionic blockade with hexamethonium caused greater fall in the BP of HS rats (33%), and central antagonism of AT_1_ receptors (losartan) microinjected into the lateral ventricle reduced BP level of HS, but not of Cont group. Heart rate variability analysis revealed sympathetic prevalence on modulation of the systolic interval. HS diet did not affect creatinine clearance. Kidney histological analysis revealed no significant change in renal corpuscle structure. Sodium and potassium concentrations in CSF were found higher in HS rats despite no change in plasma concentration of these ions. Taken together, data suggest that animals exposed to chronic salt intake to a level close to that reported for human’ diet since weaning lead to hypertension, which appears to rely on sodium-driven neurogenic mechanisms.

## Introduction

**Figure 1:**
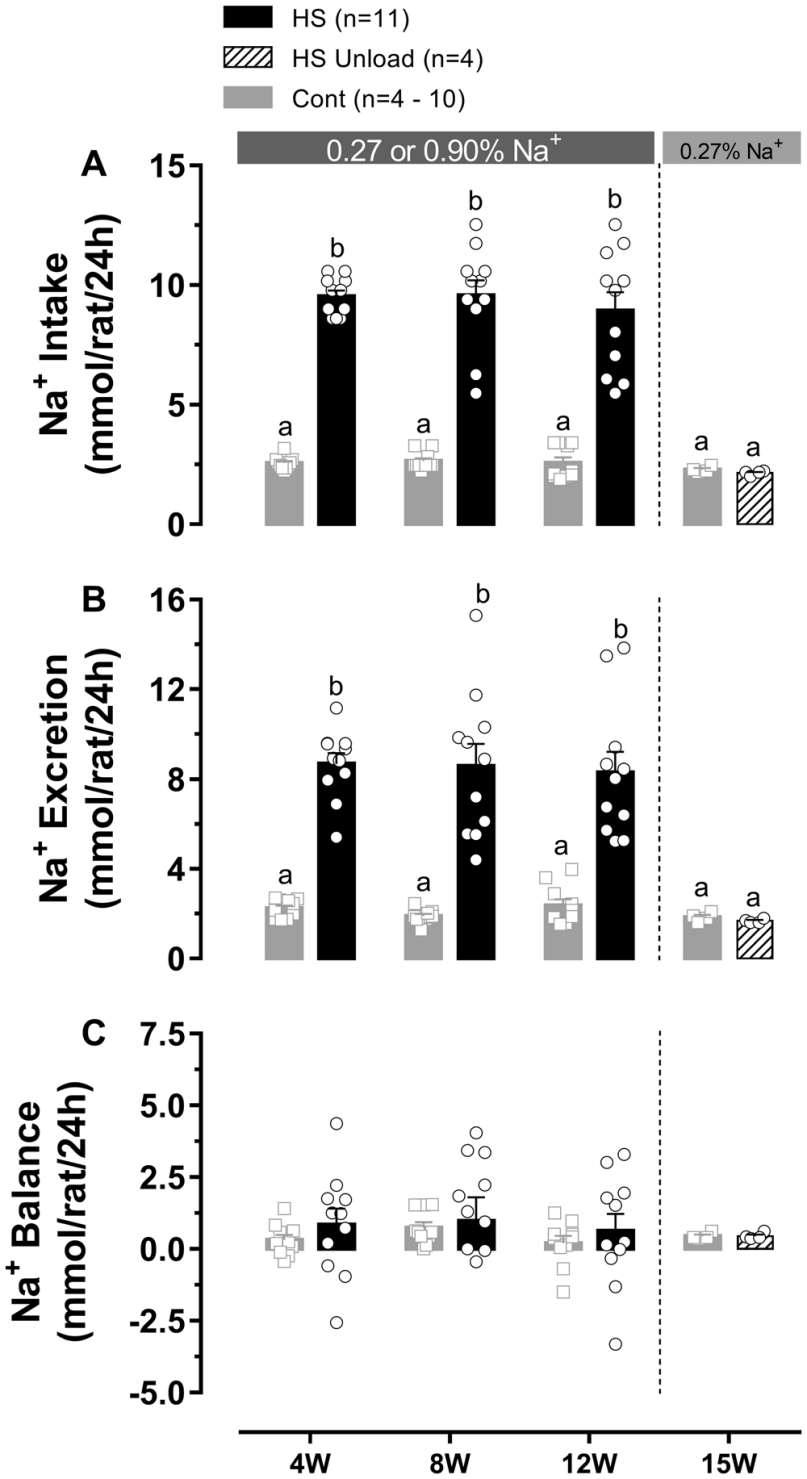
Sodium (Na) intake (**A**), sodium excretion (**B**) and 24-hours sodium balance (**C**) of rats from HS, HS Unload and respective control groups. Measurements were performed on the 4^th^ (4 W), 8^th^ (8 W), 12^th^ (12 W) and 15^th^ (15 W) weeks after weaning for 24 h in metabolic cages. Scattered squares (Cont) and circles (HS/HS Unload) represent individual values and bars represent average group data. Differences between pairs of means are indicated by different letters. Same letter means no statistical difference. Two Way ANOVA followed by Bonferroni’s post-test; p < 0.05.

Sodium is an essential nutrient and the primary cation in the extracellular fluid. It is required in a number of physiologic processes as maintenance of extracellular volume and osmolality, membrane potentials and several transmembrane transport processes. From an evolutionary perspective, sodium intake was estimated to be very low (1 g/day) in primitive cultures and remained so for thousands of years^1^. About a millennium ago, salt intake in the western countries had risen to about 5 g/day and kept raising up to 9–12 g/day in the 19^th^ century^1^. In 2012, the World Health Organization (WHO) recommended that salt intake should be kept below 5 g/day^2^ in order to avoid health problems, especially those related to the cardiovascular system. Recent trial conducted in Brazil have shown that the average salt intake in a sample population from Vitoria (East coast) is estimated in 10 g/day and a significant number of individuals ingest more than 15 g/day of salt, 3 times the recommended by the WHO (Fig. 1 of the referenced paper)^3^. High salt intake has been associated with high blood pressure (BP) since 1904, when Ambard and Beaujard first established this correlation^4^. Since then, an overwhelming number of studies have been made on this topic and linked hypertension to high-sodium intake both in humans^3^ and experimental^5–12^ models. Yet, some questions regarding salt-dependent hypertension remain elusive, in particular, those concerning whether the amount of daily sodium consumption in the diet by humans can, *per se*, elicit hypertension in rodent models.

More than a few experimental strategies have been developed to induce sodium-dependent hypertension in adult rodents and extensively used to study its origins and mechanisms^13, 14^. Adult Sprague-Dawley rats under moderate salt diet (2% NaCl) and chronic infusion of angiotensin II, which clamps circulating angiotensin II levels, develop high levels of blood pressure within days after infusion starts^15, 16^. Replacement of drinking water by sodium chloride solution results in high salt/water intake relation and also lead to hypertension in adult rats^12, 17^. One issue here is that none of the mentioned conditions are common in humans under high sodium intake and displaying high blood pressure. Another experimental approach is to exposure adult normotensive rats to diets containing very high levels of sodium chloride (4 to 8% w/w) which also results in hypertension within weeks to months of exposure^8, 18^. Some of these models use diets with salt content about 8–16 times higher than recommended for rodents in order to successfully produce high blood pressure^8, 9, 19^. These levels of diet salt are considerably higher than reported for daily salt intake by humans (2–4 folds)^3^ and thus raise some difficulties for the understanding of actual mechanisms leading to sodium-hypertension in humans. Currently, foods with high sodium content are usually introduced very early in the children’s diet and in many cases, the individual remains on a high salt diet for his entire life. Therefore, such characteristic should be considered by experimental models studying salt-dependent hypertension.

Studies using experimental animal models as well as in humans led to two main theories about the primary mechanisms causing salt-dependent hypertension: i) sodium and water retention in the extracellular fluids, which according to the Guyton’s theory of impaired renal pressure-natriuresis may explain the associated alterations in blood volume and peripheral resistance; ii) the neurogenic mechanisms that lead to sympathoexcitatory responses to different territories, and consequently to increase in BP.

In fact, several studies performed over the last decades brought upon several pieces of evidence supporting the hypothesis that salt-dependent hypertension involves (and may be initiated by) neurogenic mechanisms^15, 20–22^, which increase sympathetic outflow to peripheral blood vessels and total peripheral resistance^23^. The sympathetic drive is maintained and modulated by the activity of brain regions within the medulla and forebrain areas susceptible to changes in neurotransmission by high salt intake^13, 24–26^. However, the mechanisms by which such regions are stimulated by high-salt intake remain elusive.

**Figure 2:**
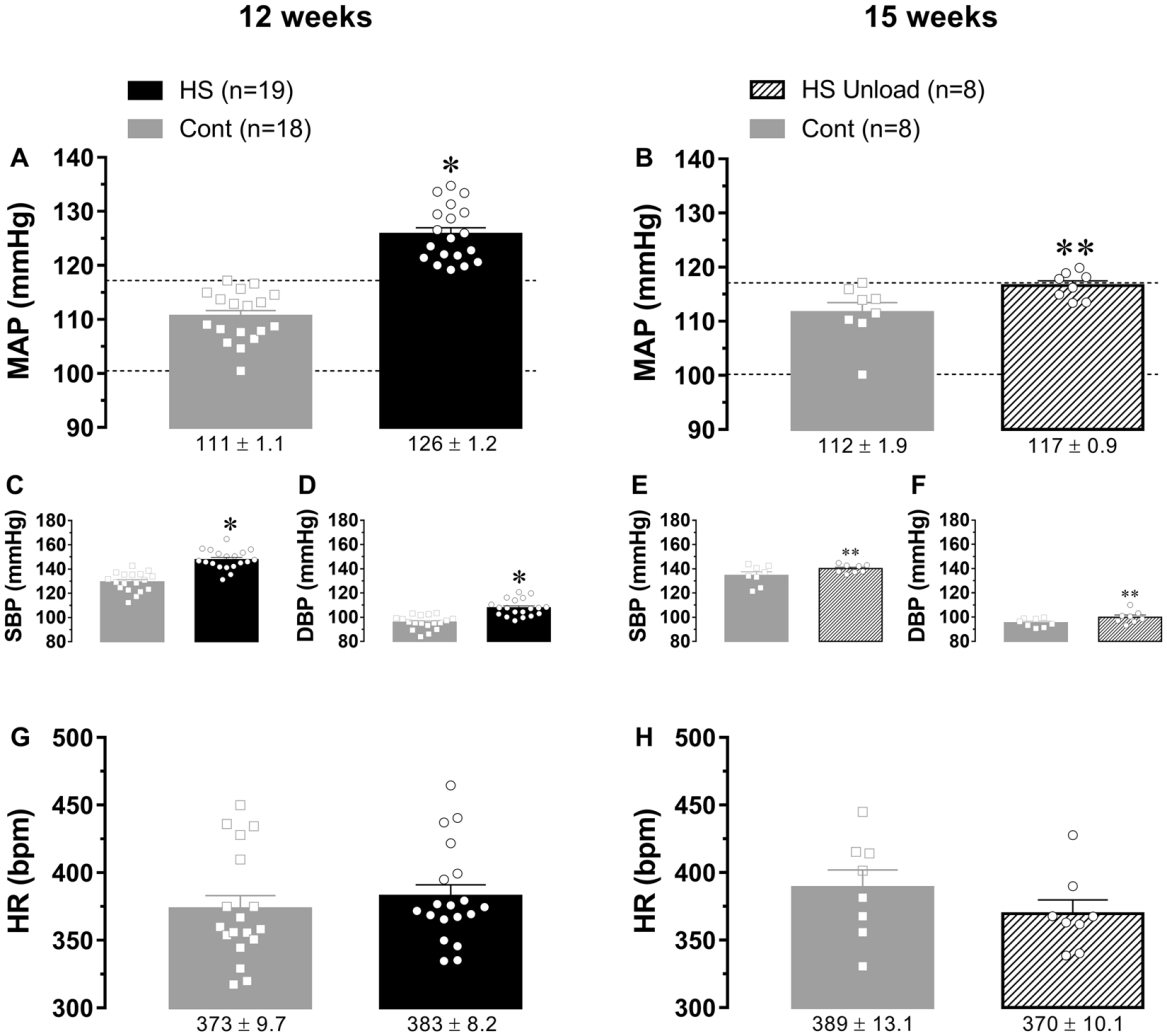
Baseline levels of mean arterial blood pressure (MAP) and heart rate (HR) in rats from HS, HS Unload and respective control groups. Direct measurements of MAP and HR were performed on the 12^th^ and 15^th^ weeks after weaning. Scattered squares (Cont) and circles (HS/HS Unload) represent individual values and bars represent average group data. Average mean ± SEM are indicated under the bottom of respective bars. Horizontal dashed lines define the boundaries for control MAP and HR range. Systolic blood pressure (SBP) and diastolic blood pressure (DBP) for all groups are shown in the smaller panels (C through F). *Different of control group; one way ANOVA followed by Bonferroni’s post-test; **different of HS group; one way ANOVA followed by Bonferroni’s post-test; p < 0.05.

Recent evidence suggested that changes in the cerebrospinal fluid (CSF) ionic composition strongly influence the control of sympathetic nerve activity and, therefore, may play a pivotal role in BP control. Increased CSF sodium levels have been documented in Dahl-S and spontaneously hypertensive-rats (SHR) under high-salt diets, correlates with high BP levels and precede hypertension in these experimental models^6^. Further, normotensive adult Wistar rats are shown to develop hypertension when high-sodium artificial CSF is infused intracerebroventricularly (*i*.*c*.*v*.) during a 14-days period^27^. Authors suggested that high blood pressure develops as a consequence of higher than normal sympathetic outflow driven by high-sodium concentration in the CSF^27^. In fact, it was recently demonstrated that ICV infusion of high-sodium artificial CSF increased lumbar and adrenal sympathetic nerve discharge as well as blood pressure^28^. Replace drink water by 1 mol/L NaCl solution for 14 days also increased sympathoexcitatory and sympathoinhibitory responses from rostral ventrolateral medulla (Figs 1 and 2 of the Adams, J. M., *et al*. paper)^7^ the major origin of pre-motor sympathetic neurons in the central nervous system^23^. Despite these compelling evidence, the cause-consequence relationship between “mild” sodium diets intake, close to that reported for humans, and the development of hypertension in experimental rodent models as well as the mechanisms involved require further studies.

Therefore, this study was designed to focus on two major questions. The first was to test whether weaned Wistar rats under ongoing daily sodium intake close to that reported for humans can develop hypertension at the adult age. The second was to evaluate whether chronic high-sodium intake can produce sympathetic dependent hypertension and if sodium concentration in the CSF would be correlated with blood pressure in rats under high-salt diet.

## Results

### Effects of high-sodium diet on growth and sodium balance

Chronic intake of high-sodium diets did not change body mass of the HS or HS Unload groups when compared to the respective Cont groups throughout the 12 weeks of treatment. Likewise, HS Unload and Cont groups had similar body weights at the end of the 15-weeks treatment (Supplementary Table 1S). Twenty-four-hours food intake was measured in metabolic cages on the 4^th^, 8^th^ and 12^th^ weeks. The three-week average food intake was not different between HS and Cont groups. Food intake in HS Unload group also was not different of that in Cont (Supplementary Table 1S).

Sodium intake was individually calculated for each rat based on food intake and sodium concentration in the chow. As expected, average sodium intake in 24 h on the week 4, 8 and 12 was higher (360%) in HS group compared to control group. On the 3^rd^ week, after HS diet was replaced by regular sodium diet, sodium intake returned to normal and reached 94% of control. Detailed group comparisons within each week are summarized in Fig. 1. The three-week average urine sodium concentration (Supplementary Figure 2S) and sodium excretion were 235% and 393% higher in rats under HS diet, respectively. At the end of 3 weeks under regular-sodium diet, urine sodium concentration (Supplementary Figure 2S), and sodium excretion returned to normal as shown for the HS Unload group. Sodium balance between groups was not different. Data are summarized in the Fig. 1A–C.

HS diet also resulted in increased water intake as well as urine output. The water balance was shown to be increased in HS rats as well. Water intake, urine output and water balance returned to normal 3 weeks after switch high-sodium to regular-sodium diet (Supplementary Figure 1S).

### High-sodium diet increases blood pressure

Wistar rats on the high-sodium diet from weaning throughout the 12-week period displayed a significant increase in resting mean arterial pressure (MAP) (126 ± 1 mmHg, n = 19) when compared to Cont group (111 ± 1 mmHg, n = 18), but no changes in the resting heart rate (HR) (Fig. 2A,G). Systolic and diastolic blood pressure levels were also high in HS rats compared to Cont rats (Fig. 2C,D). As shown in Fig. 2A and B, resting MAP of the HS Unload group on the 15th week was not different of that found in respective control group (117 ± 1 mmHg HS Unload vs. 112 ± 2 mmHg Cont; one way ANOVA followed by Bonferroni’s post-test; p = 0.2292). On the other hand, resting MAP of HS Unload group was 9 mmHg smaller than HS rats (117 ± 1 mmHg HS Unload vs. 126 ± 1 HS; one way ANOVA followed by Bonferroni’s post-test; p < 0.0002). Systolic and diastolic blood pressure levels between these two groups were not different (Fig. 2E and F).

### Salt-induced hypertension relies on a higher sympathetic drive

**Figure 3:**
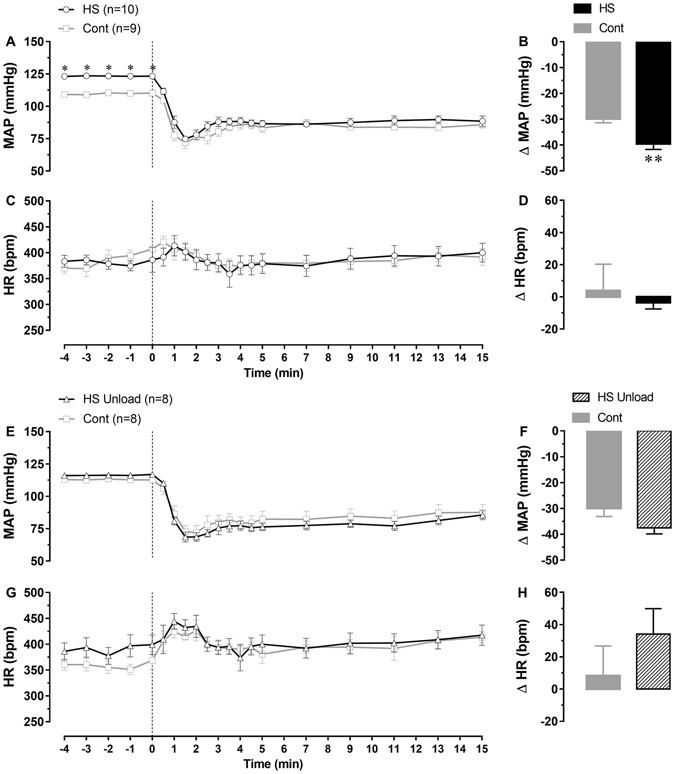
MAP and HR levels before and after hexamethonium injection in rats from HS, HS Unload and respective control groups. Experiments were carried out on the 12^th^ (for HS group) and 15^th^ (for HS Unload group) weeks after weaning. Vertical dashed lines indicate the moment of the *i*.*v*. injection of hexamethonium (20 mg/kg). Panels B,D,F,H represent the change in MAP and HR 7 to 11 minutes after the injection. *Different of control; two-way ANOVA for repeated measurements followed by Bonferroni’s post-test; p < 0.05. **Different of control; unpaired *t*-test; p < 0.05.

To assess the role of sympathetic drive in maintaining the high MAP of HS rats, hexamethonium, a ganglionic blocker, was injected intravenously (*i*.*v*.). Figure 3A shows that the fall in the MAP of HS rats were greater (ΔMAP −40 ± 2 mmHg; n = 10; unpaired *t*-test; p = 0.0026) than in Cont group (ΔMAP −30 ± 2 mmHg, n = 9, Fig. 3, panel B) following hexamethonium treatment. These results suggest greater sympathetic activation in the HS-fed rats.

For HS Unload rats, changes in MAP (ΔMAP −38 ± 2 mmHg, n = 8; unpaired *t*-test; p = 0.0710) after hexamethonium injection was not different of that in the Cont group (ΔMAP −30 ± 3 mmHg, n = 8; Fig. 3, panel F). The levels of blood pressure were also not different between groups before or after hexamethonium (Fig. 3, panels E and G; Two Way ANOVA). No significant differences in HR levels were observed after hexamethonium between the groups (Fig. 3D,H).

Time-domain variability of the HR was evaluated by the root mean square of the successive differences (RMSSD) and the results showed smaller variability in both HS and HS Unload groups compared to their respective control groups as shown in Table 1. Spectral analysis of the systolic interval showed that very low frequency (VLF) band contribution to the total power density was bigger in the HS group when compared to the Cont group. Low frequency (LF) band contribution was similar between groups and high frequency (HF) contribution was smaller leading to a higher LF/HF ratio in the HS group compared to control.

**Table 1:** Frequency and time domain variability results of the systolic interval (SI) from HS, HS-Unload and respective control groups.

	Cont 12 W		HS			Cont 15 W		HS Unload		
	Mean ± SEM	n	Mean ± SEM	n	p	Mean ± SEM	n	Mean ± SEM	n	p
HR (bpm)	383 ± 12	(9)	381 ± 11	(10)	0.8894	386 ± 16	(8)	375 ± 11	(8)	0.5674
RMSSD (ms)	5.9 ± 0.5	(9)	4.4 ± 0.4*	(10)	0.0429	6.0 ± 0.9	(8)	3.9 ± 0.3*	(8)	0.0416
VLF (% of total Power)	50 ± 4	(9)	72 ± 4*	(10)	0.0015	50 ± 9.1	(8)	70 ± 4.8	(8)	0.0692
LF (% of total Power)	13 ± 1.3	(9)	12 ± 1.6	(10)	0.7011	16 ± 3.2	(8)	15 ± 2.3	(8)	0.7776
HF (% of total Power)	31 ± 3.3	(9)	14 ± 3.2*	(10)	0.0014	31 ± 7.0	(8)	13 ± 2.9*	(8)	0.0348
LF/HF ratio	0.46 ± 0.07	(9)	1.30 ± 0.33*	(10)	0.0316	0.74 ± 0.19	(8)	1.32 ± 0.17*	(8)	0.0413

SI data were acquired from 4,000 to 4,500 consecutive heart beats using the pulsatile blood pressure signal (approximately 10 minutes of continuous recording). Very low frequency (VLF), low frequency (LF) and high frequency (HF) bands ranged from 0.0 to 0.2 Hz, 0.2 to 0.75 Hz and 0.75 to 2.50 Hz respectively. RMSSD = root mean square of the successive differences was used to assess time-domain heart rate (HR) variability. Total Power density was measured as µs^2^. *Different of respective control group; unpaired *t*-test; p < 0.05.

For the HS Unload rats, HF band contribution to the total power density was also smaller compared to control what also led to a higher LF/HF ratio in the HS Unload group. Together, these data also suggest prevailing sympathetic modulation on the heart rate variability. The entire data set is shown in the Table 1.

### Hypertension secondary to high-sodium diet involves central activation of AT_1_ receptors

**Figure 4:**
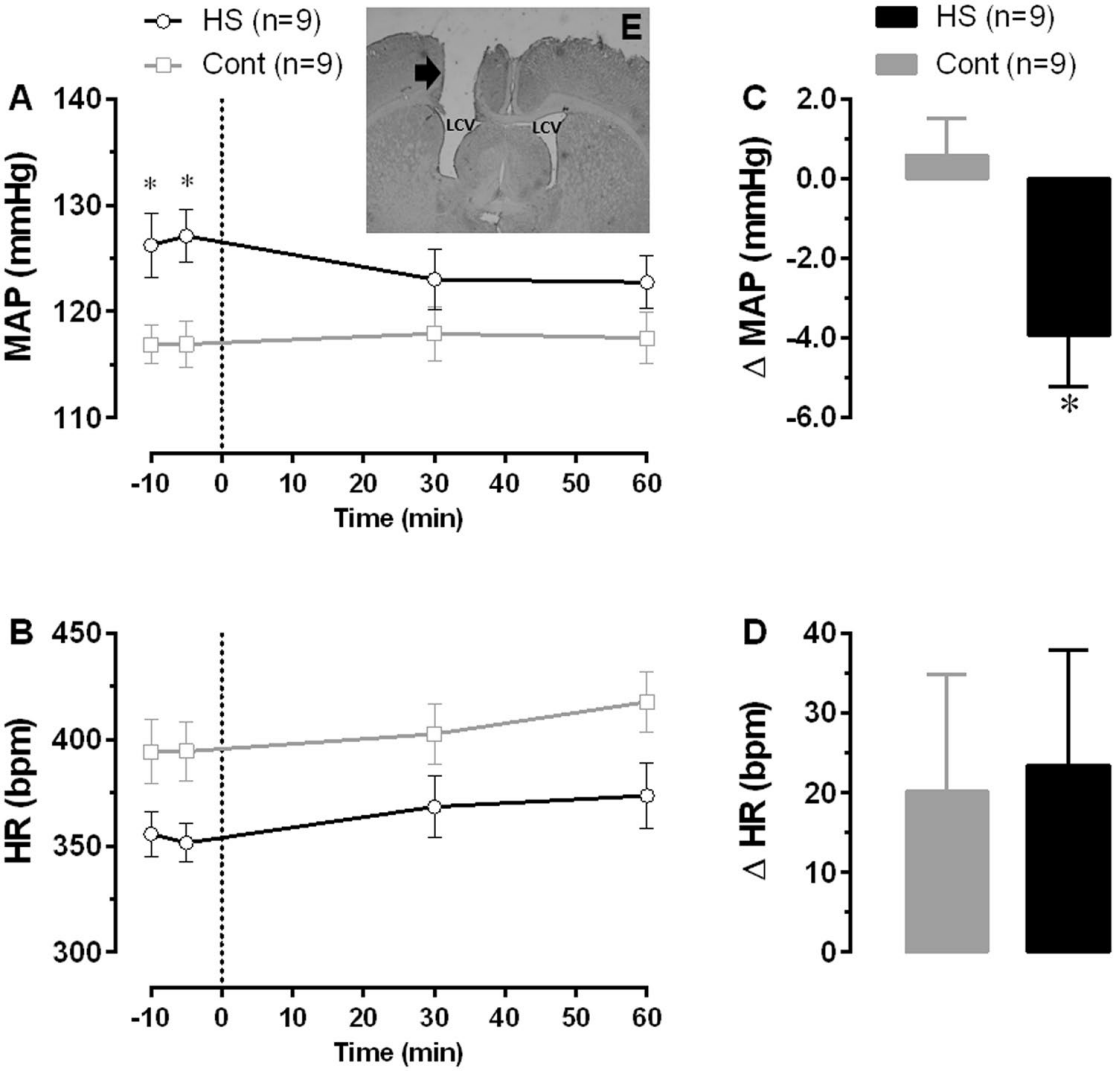
Cardiovascular effects of central losartan microinjection into the lateral cerebral ventricle (LCV). Direct measurements of blood pressure were performed on the 12^th^ week after weaning in rats from HS and respective control groups. Vertical dashed line mark the losartan microinjection (180 nmol/kg) event. Panels A and B represent absolute levels of MAP and HR over time and panels C and D represent change of MAP and HR 60 minutes after injection. Panel E shows a coronal brain slice at the lateral cerebral ventricle (LCV) level with the guide cannula tract (black arrow) indicating microinjection into the LCV. *Different of Cont group; two-way ANOVA for repeated measurements followed by Bonferroni’s post-test; p < 0.05.

In order to control for possible effects of potassium from the losartan salt, an equimolar (to losartan solution) solution of KCl dissolved in PBS was first injected into the lateral cerebral ventricle (LCV). No significant changes in MAP (ΔMAP −1 ± 0.8 mmHg for HS vs. ΔMAP −1 ± 0.9 mmHg for Cont) or HR (ΔHR −8 ± 9 bpm for HS vs. ΔHR 13 ± 16 bpm for Cont) relative to baseline was detected between groups. Figure 4C shows that central antagonism of AT_1_ receptors with losartan elicit a minor, but significant fall in MAP of the HS rats (ΔMAP −4 ± 1 mmHg, n = 9, p < 0.05) relative to baseline levels (before the injection), and when compared to the Cont group (ΔMAP 1 ± 1 mmHg, n = 9) at the same time course evaluated. No significant changes were found in HR after losartan (Fig. 4B and D).

### High-sodium diet increases Na^+^ concentration at the brain level

**Figure 5:**
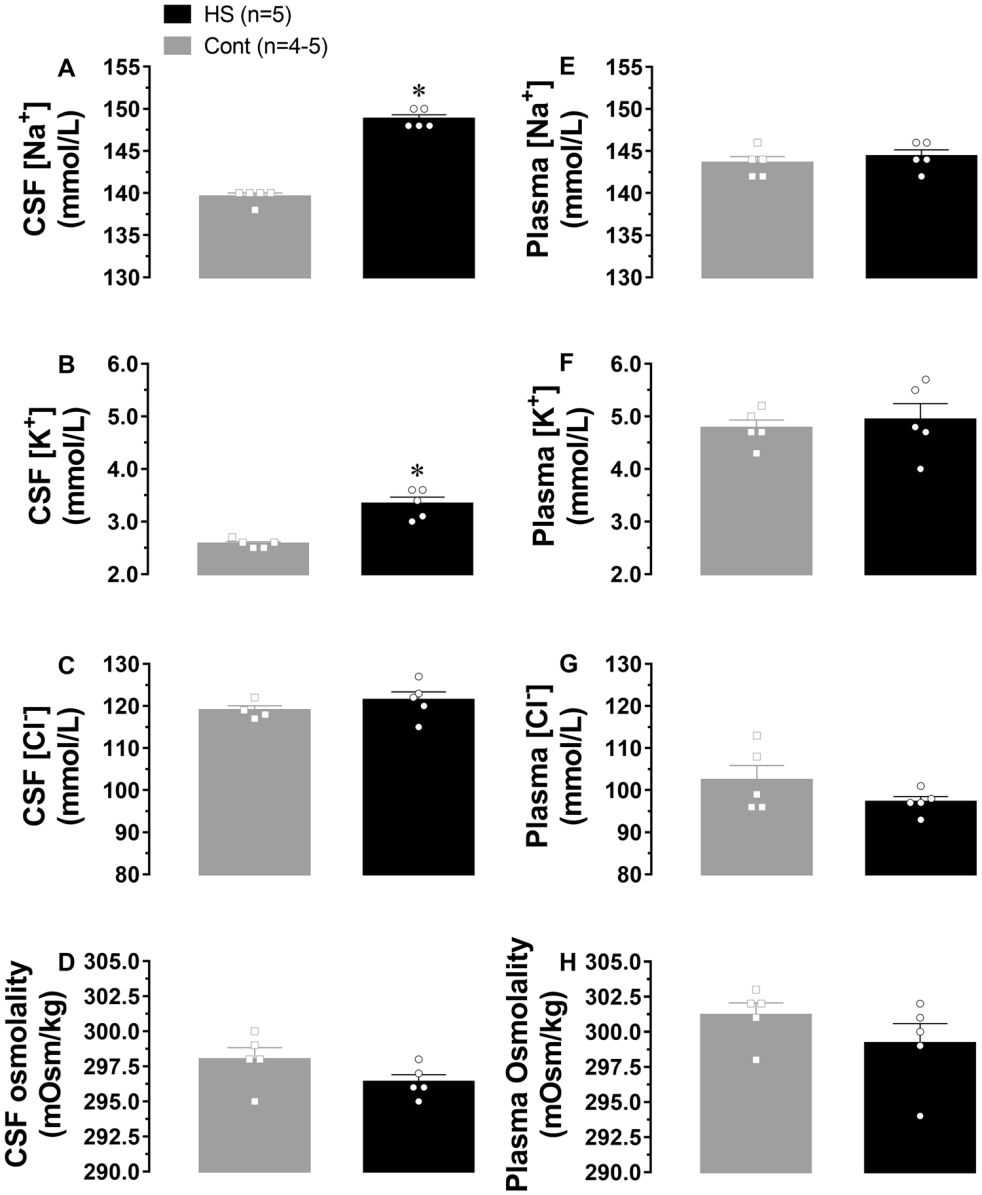
CSF and plasma concentration of sodium [Na^+^], potassium [K^+^], chloride [Cl^−^] and osmolality. Panels A and E represent [Na^+^] in CSF and pla*s*ma; (**B** and **F**) represent [K^+^] in CSF and pla*s*ma; (**C** and **G**) represent [Cl^−^] concentration in CSF and pla*s*ma and (**D** and **H**) represent osmolality measured in fresh samples. Measurements were performed in samples from HS and Cont rats on the 12^th^ weeks after weaning. *Different of Cont group; unpaired *t*-test; p < 0.05.

High-sodium diet intake did not change plasma sodium and potassium concentrations as well as plasma osmolality during the day period (Supplementary Table 2S). However, sodium and potassium concentrations in the CSF were significantly increased by 6.5% (difference of 9 mmol/L; unpaired *t*-test; p < 0.0001) and 29% (difference of 0.76 mmol/L; unpaired *t*-test; p = 0.0004), respectively, during the day period in 12 h fasted animals (Fig. 5). CSF chloride concentrations, as well as CSF osmolality (measurements in fresh samples), were not different between HS and control rats (Fig. 5). The same measurements performed in corresponding plasma samples were not different between groups (Fig. 5). CSF samples were also taken from HS-Unload and respective control rats for sodium concentration measurements. Sodium concentrations (147 ± 4.8 mmol/L for Cont, n = 4 and 138 ± 9.4 mmol/L for HS Unload, n = 4; unpaired *t*-test; p = 0.4258) and potassium concentrations (2.85 ± 0.14 mmol/L for Cont, n = 4 and 2.88 ± 0.21 mmol/L for HS Unload, n = 4; unpaired *t*-test; p = 0.9259) in CSF samples were not different between this two groups.

### High-sodium diet did not affect renal function and morphological parameters

The level of plasma sodium, potassium, albumin, urea, creatinine and osmolality, as well urine urea, creatinine and osmolality were evaluated in rats from HS and control group (Supplementary Table 2S). Results did not reveal significant changes, except for urinary urea concentration which was 22% lower in HS compared to Cont group. Creatinine clearance was used as an index for renal function and no significant change was found between groups.

**Figure 6:**
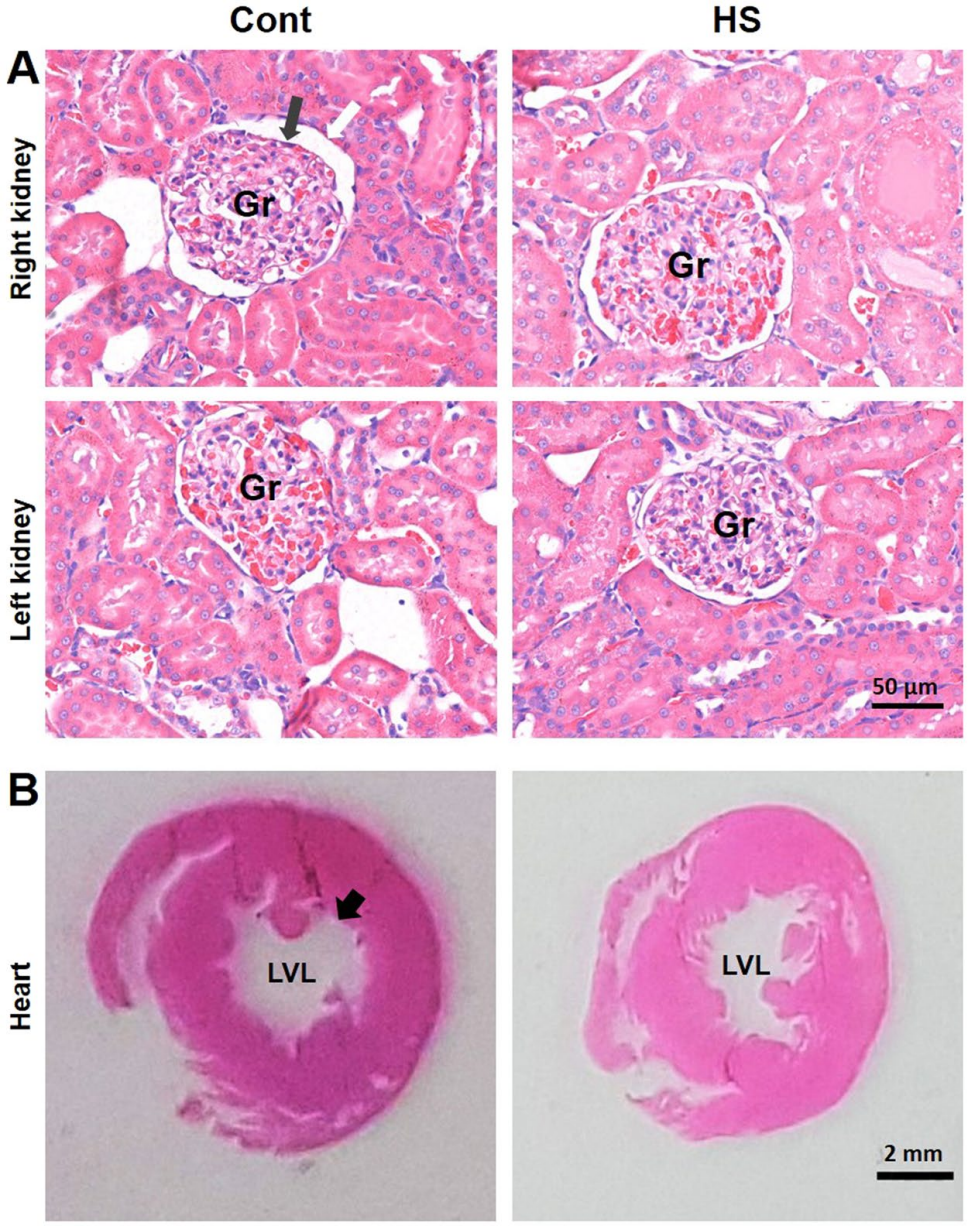
Photomicrograph histological section of the kidneys and heart from rats fed with regular (Cont) or high-sodium (HS) diet. Panel A: cortical areas of the kidneys were evaluated and renal corpuscles analysed. Grey and white arrows point to the edges of the glomerular tuft (Gr) and Bowman’s capsule respectively. No inflammatory process was detected in the kidneys sections of both groups. Kidney photomicrographs were taken using 400x magnification (bar = 50 µm). Panel B: transversal cut of the hearts taken using 1x magnification ﻿(bar = 2 mm). Black arrow point to the edge of the left ventricle lumen (LVL).

In addition, histological analysis of kidneys sections was performed to evaluate possible tissue damage, remodelling or inflammatory process as a result of high-sodium intake. As depicted in Table 2, none of the indexes used to assess glomerulosclerosis neither relative size of the kidneys was different between groups. Besides that, no inflammatory process in the kidneys was detected (Fig. 6A). In order to verify possible heart hypertrophy in hypertensive HS rats, we also evaluated the ratio between left ventricle wall thickness (Wt) and the left ventricle lumen diameter (LVL), and no significant difference was found between groups as shown in Table 2 and Fig. 6B.

**Table 2:** Morphometric analysis of renal and cardiac parameters in HS rats and respective control group.

Kidneys	Cont		HS		
	Mean ± SEM	n	Mean ± SEM	n	p
Left kidney wet weight/body weight (mg/g)	3.30 ± 0.13	(8)	3.24 ± 0.05	(8)	0.6842
Right kidney wet weight/body weight (mg/g)	3.23 ± 0.09	(8)	3.29 ± 0.07	(8)	0.6323
Sclerotic glomeruli left kidney (%)	6.25 ± 0.81	(8)	5.62 ± 0.62	(8)	0,5057
Sclerotic glomeruli right kidney (%)	7.50 ± 1.33	(8)	6.25 ± 1.25	(8)	0,5536
Left kidney BCA/GTA (µm^2^/µm^2^)	1.29 ± 0.02	(8)	1.28 ± 0.02	(8)	0.6003
Right kidney BCA/GTA (µm^2^/µm^2^)	1.30 ± 0.01	(8)	1.27 ± 0.02	(8)	0.2608
**Heart**					
Heart wet weight/body weight (mg/g)	2.72 ± 0.06	(8)	2.61 ± 0.03	(8)	0.1536
Left Wt/LVL ratio	0.77 ± 0.05	(8)	0.83 ± 0.04	(8)	0.4013

Ratios and indexes were calculated as described in the methods section. BCA/GTA = Bowman’s capsule area/glomerular tuft area; Wt/LVL = wall thickness/lumen. Pairs of means were compared by unpaired *t*-test. SEM = standard error mean.

## Discussion

Here we have shown that weaned rats fed 0.9% sodium diet (0.9%, Na^+^) until young-adult age became neurogenic-mediated hypertensive due to a higher sympathetic activity. The 0.9% sodium diet is equivalent to 2% NaCl diet and the amount of sodium in this diet is 3.3 times higher than that found in our regular rat chow (0.27%, Na^+^). Yet, the previous study using the gold standard method of 24-h urine collection to measure sodium consumption in humans have established a correlation of high systolic blood pressure with sodium chloride intake higher than 3 times the recommended by the WHO^3^, which guided our choice for a 0.9% Na^+^ content in our high sodium diet. In addition, the tonic activation of central AT_1_ receptors contributes for high BP suggesting that central mechanisms activated by angiotensin II at the brain level also play an important role in this pathology. One major finding here is that rodents under chronic moderate sodium intake displayed CSF sodium and potassium concentrations higher than those found in control rats despite no change in these ions concentrations at the plasma level. This finding suggests a correlation between salt intake, CSF sodium and potassium levels and high blood pressure.

Most of the experimental models of salt-dependent hypertension require strict experimental conditions or salt-loading condition overestimated in comparison to the salt contend in human’s diet. To bypass those limitations, we proposed a model in which the sodium intake by rats was close to that estimated for humans. The growth pattern and food intake suggest that the amount of sodium added to the diet (Na^+^ 0.9%) did not effect on overall nutrition of the rats. Moreover, our data also show that replacement of HS diet by regular-sodium diet did not affect food intake as evaluated on the 15^th^ week. Therefore, the 12 weeks of HS diet may have little effect on mechanism regulating food intake in this model. It has been shown elsewhere that weaned rats drinking saline solution for water over 8 weeks^12^ ate and grew less than respective controls. One may conclude that such approach may compromise overall animal nutrition and bring upon other hypertensive factors since previous studies have proposed that protein restriction can result in cardiovascular dysfunctions^12^. Here, rats were exposed to sodium overload just after weaning, which is a crucial period of growth and maturation of several different physiological systems. The model was designed on purpose so that we could match the diet condition of humans with habits of high-salt food consumption.

The fact that water intake was higher in HS rats suggest that 0.9% sodium diet is stimulating thirst so that extra sodium could be diluted to isotonic concentrations in the rat body^29^. High urine output, urine sodium concentration and sodium excretion strongly suggest that extra sodium have been excreted within a larger urine volume. Consequently, sodium balance was kept close to normal among groups as shown in Fig. 1. Together with no changes in the plasma sodium level, osmolality and albumin concentration our data suggested that extra sodium is not accumulating in the peripheral extracellular fluid to produce hypervolemia. These fundamental aspects of our model have important clinical and physiological implications because most of the extra sodium ingested has been excreted in the urine mimicking physiological conditions of humans ingesting HS diets.

Increased plasma sodium concentration is known to reduce renin secretion, blood borne angiotensin II and, therefore, sodium reabsorption by the kidneys in order to maintain the plasma sodium levels within narrow limits of variation^29^. To produce hypertension in adult rats under diets with sodium content close to what humans ingest (2% NaCl), clamping angiotensin II levels is required for rodents. This approach has been extensively used and is called AngII-salt model^15^. Our results clearly show that Wistar rats under 0.9% sodium (2% NaCl) diet from 21 days to 12 weeks after weaning, i.e. 15 weeks of age, significantly increased mean arterial pressure without the need for clamping circulating angiotensin II levels. Therefore, adaptations in the kidney renin secretion controlling mechanisms may take place to prevent blood born angiotensin II depletion in this model. Conversely to our results, Ufnal *et al*.^30^ found that rats fed 0.82% sodium diet (close to the sodium levels we used in our experimental model) for 8 weeks after weaning did not increase BP levels, even when diet sodium content raised to 3.28%. These findings may suggest that sodium-dependent hypertension seems to depend mostly on time of exposure and less on the magnitude of sodium overload. This would explain why these authors have not found changes in BP with 8 weeks of HS diet, while our data show that 100% of rats fed HS diet increased BP with no significant change in HR.

Previous studies have shown that 2% NaCl (0.9% Na^+^) diet over a period of 2 weeks did not produce any significant increase in BP of normotensive adult male Sprague-Dawley rats^15^. Based on this data, we speculate that adult rats could handle the 0.9% sodium overload in such a way that rises in BP would not be required to produce pressure-dependent natriuresis as proposed by Guyton and Coleman in 1972^31^. If the pressure-dependent natriuresis model is taken as a starting point, without considering neurogenic mechanisms, the increased BP could be interpreted as a result of kidney damage due to chronic exposure to sodium overload. Preceding data have shown that very high sodium loading (8% NaCl) in the diet for 8 weeks after weaning can, in fact, result in more severe hypertension, secondary and strongly correlated with renal function deterioration as indexed by proteinuria^8^. In this scenario, increased BP might be required to maintain sodium excretion and, therefore, sodium balance. To test this hypothesis, we evaluate creatinine clearance and urea concentration both in the plasma and urine. Our data clearly show that renal function of rats fed with HS diet seem to operate within a normal range, since creatinine clearance, plasma urea concentrations and sodium balance were not different of that found in rats under regular-sodium diet. Urine low urea concentration is most likely the result of increased urinary flow of rats fed HS diet, further diluting urea in a bigger urine volume. Additionally, histological analysis of renal corpuscle structures also suggested that glomerulosclerosis indexes in rats fed with HS diet are similar to those found in control rats, countering the hypothesis that BP rises as a consequence of renal damage. Also, left ventricle wall thickness and lumen diameter ratio were not different between Cont and HS rats suggesting that 0.9% sodium diet did not produce heart hypertrophy over the 12 weeks of high-sodium diet exposure. Therefore, a neurogenic origin for hypertension reported in this study, as proposed in the literature for other salt-dependent hypertension models^14^, seem to be the most likely candidate to explain the high levels of BP.

The precise neurogenic mechanisms that operate in salt-dependent hypertension are not fully understood. However, several studies have been made valuable contributions to better understand it, as such: (1) 2% sodium chloride diet (approximately 0.9% sodium) combined with angiotensin II infusion produced hypertension, which is largely dependent on splanchnic sympathetic drive^15^; (2) excess dietary NaCl intake for 14 days produced greater splanchnic sympathetic responses to the exogenous excitatory neurotransmitter l-glutamate and inhibitory neurotransmitter GABA^7^ and alters the angiotensinergic regulation of neurons^32^ in the rostral ventral lateral medulla (RVLM), a bulbspinal cluster of neurons that provides most of the excitation to sympathetic preganglionic vasomotor neurons that convey sympathetic drive to the cardiovascular system^23, 33^; (3) the organum vasculosum of the lamina terminalis (OVLT) lesion markedly attenuated BP elevation produced by angiotensin II infusion in rats on 2% NaCl diet suggesting that the central nervous system holds the major target for the pressor mechanisms activated in the angiotensin II-salt dependent hypertension model^34^. Such evidence strongly suggest that central nervous system plays a major role in the pathogenesis of sodium-dependent hypertension. Hence, we decided to explore possible neurogenic mechanisms operating in our model of HS dependent hypertension. As a first approach, the ganglionic blockade was performed as an experimental approach also been used in classic studies of the human essential hypertension^26^. We are able to show that HS-diet induced hypertension is dependent on a sympathetic drive because HS animals had a greater fall in the BP level after ganglionic blockade. Moreover, the BP felt to the same level in HS and Cont groups suggesting that vasoconstriction by hormonal mechanisms or increased plasma volume might not be the primary hypertensive drive in our model.

The analysis of the systolic interval revealed an increase in the VLF component contributing to the total power density in HS group what may be interpreted as a greater contribution of some humoral factors to the total HR variability^35^ of HS fed rats. More detailed studies are required to further our understanding of such humoral factors in salt-dependent hypertension. Frequency-domain variability analysis also showed that LF/HF ratio was higher in the HS group what may be interpreted as an increased contribution of sympathetic modulation to the HR variability^36, 37^. However, some caution must be exercised when interpreting this data. Results clearly showed that LF band was similar between HS and Cont groups while HF band was smaller in the HS group. Therefore, LF/HF ratio was higher in the HS groups not because sympathetic modulation increased, but because HF component was reduced in the HS group probably reflecting diminished respiratory sinus arrhythmia, which is linked to parasympathetic cardiovagal outflow^38^. This matter has been reviewed recently in the literature, and since LF band was similar between HS and Cont groups, HS intake may not be affecting baroreflex modulation of BP, but changing the sympathovagal balance and bias the system in favour of higher sympathetic contribution^38^. Time-domain variability evaluated ty RMSSD was smaller in HS and HS Unload rats corroborate the idea of reduced respiratory sinus arrhythmia due to high-sodium intake because RMSSD also can be linked to such phenomenon^38^. When high-sodium diet was replaced by regular-sodium diet, resting BP decreased to levels close to that found in control rats, and ganglionic blockade no longer produced a significant greater effect. Altogether, these findings indicate that sympathetic drive seems to be the most important ongoing mechanism sustaining high BP in Wistar rats fed with HS diet.

We speculate that such increase in sympathetic drive originates from neurochemical changes at the autonomic brain nuclei regulating blood pressure. Some of these free blood-brain barrier nuclei present AT_1_-type receptors, densely distributed in the lamina terminalis (LT), OVLT and subfornical organ (SFO), and more moderately in the area postrema (AP)^39, 40^. To evaluate whether forebrain pathways activated by angiotensin II were involved with BP elevation in HS rats, we performed a central antagonism of AT_1_ receptors with losartan, and the results showed a minor, but significant decrease in the BP of HS rats after the antagonism of AT_1_ receptors, indicating that losartan is acting on this receptors in these regions. The small reduction in BP due to the antagonism of central AT_1_ receptors suggests that other mechanisms operate along with angiotensin II.

Many studies have reported a significant increase in sympathetic drive and BP after acute increasing in the CSF sodium concentration suggesting the existence of a sodium-sensing mechanism that increases ongoing sympathetic activity^6, 11, 27, 28, 41^. Direct sodium-sensing mechanisms seem to regulate sodium intake through a specific membrane channel, the so called Na_x_ channels, which are expressed in the OVLT and SFO^41^. The OVLT is located in the LT, lack a complete blood-brain barrier, is activated by hypernatremia^42–44^ and connect to other regions in the LT^44^. The main downstream targets of projecting neurons in the LT is the paraventricular nucleus of the hypothalamus (PVN) which modulates sympathetic nerve activity under certain conditions, and increase sympathetic drive when plasma osmotic or sodium stimuli activate the LT-PVN pathway^45^. Extracellular sodium concentration activates OVLT neurons *in vitro* ^46^ and since a sodium-sensing mechanism seems to be present in the OVLT as well, one can assume that increased sodium in the brain could be sensed by neurons within the OVLT and chronic augment ongoing sympathetic drive and blood pressure. This hypothesis could provide a reasonable explanation of why the AT_1_ blockade was not able to further decrease blood pressure in HS rats.

Conversely, other studies have also proposed that HS intake induces hypertension through osmotic sensing mechanisms that activate sympathetic pathways^42, 44, 45, 47, 48^. However, we have not found steady-state increased plasma osmolality in HS rats. This could indicate that peripheral osmotic sensing mechanisms are not continuously operating to support increased sympathetic drive in our model. Perusing evidence to support the idea that sodium intake might, somehow, stimulate central mechanism controlling sympathetic drive, we measured Na^+^ and K^+^ concentrations in the CSF and found them higher than normal in rats fed 0.9% sodium diet. Increased sodium level in the CSF of rats under HS diets (4 to 8% NaCl) was reported in Dahl-S and SHR^6, 49^, but never in Wistar rats fed 0.9% sodium diet. Increased sodium concentration in the CSF was also reported in humans under HS diets^50^, and may be a key element to producing increased sympathetic drive to the cardiovascular system. The choroid plexus regulates potassium concentration in the CSF by a variety of channels and ion exchangers keeping potassium concentration in the CSF about the half of the concentrations found in plasma^51^. Our data showed that potassium concentration in the CSF of HS rats increase by 29% and sodium concentration increased by 6.5% when compared to Cont group suggesting that changes in membrane conductance for potassium through the choroid plexus might also affect CSF sodium concentration^51^. This condition could lead to an electrochemical gradient change in the brain that allows higher neuronal activity in regions controlling sympathetic output. This hypothesis is consistent with recent studies which showed increased BP and sympathetic drive when CSF sodium concentrations are elevated^27, 28^. In the same way, studies performed by Osborn *et al*. in 2014 have demonstrated that *i*.*c*.*v*. infusion of benzamil, a Na^+^ channel blocker, prevented the neurogenic phase of the AngII-salt hypertension model^25^. Authors discussed that benzamil could be blocking sodium conductance in the choroid plexus, and then allow sodium concentration to fall in the CSF to reduce sympathetic drive by osmotic (or sodium) stimuli^25^. Our results also showed no change in CSF chloride concentration or CSF osmolality. Although an increased in CSF sodium concentration is expected to cause increased CSF osmolality, recent studies have reported that osmolality and sodium concentration in skin extracellular fluids versus plasma of high-salt fed rats may change differentially, and not directly correlate^52^. We don’t know whether the increase of extracellular potassium is contributing to high-salt-induced hypertension, but is plausible to speculate that it could also be accounting for higher than normal neuronal activity and, consequently, for increased sympathetic drive in HS fed rats.

In summary, our results suggested that high BP in our salt-dependent model of hypertension might result from a neurogenic mechanism influenced by increased sodium and potassium content in the brain because of animals exposed to HS diet intake since they weaned. This study showed that diet sodium intake at the level close to that reported for human’s can elicit hypertension in Wistar rats, which seems to be initiated by neurogenic mechanisms. We foresee that such model may add some pieces on the complex puzzle of elements involved in sodium-related hypertension, and reveal new strategies to treat this pathology in the future.

## Material and Methods

### Animals

All experimental protocols were evaluated and approved by the Institutional Animal Care and Use Committee (CEUA/UFOP, certificate #2013/65 and #2015/18) and were conducted in accordance with Brazilian College of Animal guidelines. Male Wistar rats 21 days old (35–50 g) were obtained from institutional animal facility and randomly divided into three cohorts: 1) high salt-group (HS) that received high-sodium (0.90% sodium w/w) powdered chow (NuviLab, Paulínea, Brazil) for 12 weeks after weaning, n = 34; 2) regular salt-group (Cont) that received regular-sodium (0.27% sodium w/w) powdered chow for 12 weeks after weaning, n = 33; 3) high-salt unload group (HS Unload), in which the animals were provided with regular-sodium diet additional 3 weeks after 12 weeks of exposure to the high-sodium diet, n = 8; 4) regular salt-group (Cont) that received regular-sodium (0.27% sodium w/w) powdered chow for 15 weeks after weaning, n = 8. High-sodium diet was obtained by adding sodium chloride to the regular-sodium chow up to the final concentration of 0.90% sodium w/w. All rats were housed in a animal facility under controlled temperature (22–24 °C), humidity (40–60%), light:dark cycle (12 h:12 h) and provided with free access to tap water and respective diet *ad libitum* for 12 (HS and respective control) or 15 weeks (HS Unload and respective control).

### Food, water intake and urine output measurements

On the week 4, 8, 12 and 15 after weaning, rats were weighed and individually housed in metabolic cages (Tecniplast SPA) for a single period of 48 hours and provided with tap water and regular or high-salt chow *ad libitum*. Urine output and water intake were measured gravimetrically within the last 24 hours in the metabolic cage (supplementary information). Food intake was calculated and expressed as amount/24 hours (supplementary information). Sodium intake was calculated multiplying the 24-hours food intake by the sodium content in each experimental diet (0.117 mmol/g in the regular diet and 0.391 mmol/g in the high sodium diet).

### Cardiovascular measurements

Within 3 days after the 12 weeks under regular or high-sodium diets, rats were anesthetized with a mixture of ketamine (80 mg/kg, *i*.*p*.; Syntec do Brasil Ltda, Hortolândia, SP) and xylazine (7 mg/kg, *i*.*p*.; Syntec do Brasil Ltda., Hortolândia, SP). Polyethylene catheters (PE-10 connected to PE-50, Clay Adams, Parsippany, NJ, USA) filled with heparinized saline (500 IU/mL) were inserted into the femoral artery and vein for measurement of pulsatile arterial pressure (PAP), and drug injections, respectively as detailed described elsewhere^53^. Blood pressure was recorded at 1000 Hz sampling rate and 20 mV range digitizing window. HR and MAP were derived on-line from the pulsatile arterial pressure signal with the LabChart 8.1.5 for Windows software (ADInstruments Pty Ltd, Australia). Experimental protocols were carried out in anaesthetized freely moving rats.

### Intracerebral injections

A subgroup of Cont rats (n = 9) and HS (n = 9) rats underwent a stereotaxic surgery for microinjection of losartan (180 nmol/kg) into the LCV. Before anaesthesia, rats received a dose of the analgesic and anti-inflammatory ketoprofen (2 mg/kg, *i*.*m*.). Under ketamine (80 mg/kg, *i*.*p*.; Syntec do Brasil Ltda, Hortolândia, SP) plus xylazine (7 mg/kg, *i*.*p*.; Syntec do Brasil Ltda, Hortolândia, SP) anaesthesia, rats were placed in a stereotaxic head frame (Stoelting Co., Illinois, EUA) for implant of stainless guide cannula directed to the LCV as previously described^54^. Upon recovery from anaesthesia, rats were individually housed in separated cages for a 3-days recovery period with free access to tap water and regular or high-sodium powdered chow *ad libitum*. Afterward, surgery for catheter’s implantation was also performed as described above and experimental protocols carried out 48 hours thereafter.

#### Experiment 1 – ganglionic blockade

Resting MAP and HR were recorded in freely moving rats during 30 min after allowing the rat to acclimatize in the experimental room for at least 45 min. The ganglionic blocker, hexamethonium (20 mg/kg), an antagonist of the nicotinic cholinergic receptors, was injected *i*.*v*. over 1 min period and cardiovascular parameters were recorded for additional 20 min.

#### Experiment 2 – antagonism of AT_1_ receptors

Central injections of potassium chloride solution in PBS (180 nmol/kg; 65 nmol/µL) as vehicle control were performed *i*.*c*.*v*. and followed by losartan (180 nmol/kg; 65 nmol/µL) microinjection *i*.*c*.*v*. 1 hour after in conscious and freely moving rats. Microinjections were made using a 5 μl Hamilton syringe connected by polyethylene tubing (PE-10) to an injector needle inserted into the LCV through the guide cannula. Each 1 µL volume injection was delivered within a period of 30 seconds. At the end of each experiment, injection site was marked with 2% Evan’s Blue solution (1 µL). Animals were deeply anesthetized with an overdose of sodium thiopental (100 mg/kg of body weight, *i*.*v*.) and perfused with 10% buffered formalin. Brains were removed and kept in 10% buffered formalin solution until histological analysis. Coronal slices were cut to a thickness of 50 μm on a freezing cryostat, mounted on glass slides, stained with cresyl violet and analysed under light microscopy to determine the site of injection. Only rats in which injection site was confirmed to be in the lateral ventricle were included in the analysis.

### CSF, blood and tissue sampling

For CSF and blood sampling, 12 hours fasted animals were anesthetized with isoflurane. In a stereotaxic apparatus, a group of 10 animals (5 HS and 5 Cont) had the occipital muscles gently removed to reach the atlantooccipital membrane. A 30 G needle was inserted into the cisterna magna and 100 µL of CSF was taken. Osmolality was immediately measured. A second part of the sample was frozen in liquid nitrogen for further analysis of sodium, potassium and chloride concentrations. Blood samples were then taken and immediately centrifuged. Plasma was separated and osmolality immediately measured. A second part was frozen in liquid nitrogen for sodium, potassium and chloride concentration measures. For biochemical analysis, a separate group of 10 HS rats and 10 Cont rats were used. Heart and kidneys were excised from 8 rats of each group, weighted and fixed in 10% neutral-buffered formalin solution until tissue processing. Blood samples were taken, immediately centrifuged and plasma samples were frozen until biochemical analysis.

### Heart and kidney histology

Morphometric analyses were based on techniques described elsewhere^55^. Briefly, hearts and kidneys were dehydrated, cleared, and embedded in paraffin. Paraffin blocks were cut into 4–5-μm thick sections, and adjacent sections were stained with haematoxylin/eosin for evaluation of general renal and myocardial morphology and identification of possible tissue damage. All morphometric measurements were made in tissue sections under a light microscope (Leica DM5000) and analysed using the Leica Qwin Image Processing and Analysis Software (Germany). To assess high-sodium diet effect on heart, the left ventricle Wt and left ventricle lumen diameter (LVL) were determined on sections at 1x magnification^56^. Cuts (3 to 5 sections) were performed at the same level of the hearts, transversally and perpendicularly with the longitudinal axis, at the middle level of the ventricles where the ventricles had bigger diameter. The degree of cardiac hypertrophy was calculated as the ratio between left ventricle Wt and LVL (see supplementary data). In order to evaluate the extent of renal damage imposed by the high-sodium diet, glomerulosclerosis was assessed by counting the number of sclerotic glomeruli and express it as the percent of total glomeruli counted in a sequence of 25 field and by the ratio between glomerular tuft area (GTA) and Bowman’s capsule area (BCA)^56^.

### Biochemical analysis

Creatinine and urea concentrations were measured in urine samples and albumin, creatinine and urea concentrations were measured in plasma samples (supplementary information). Plasma and CSF osmolality were measured in a freezing point micro osmometer (µOsmetteTM, Precision Systems), just after withdrawing. Sodium and potassium concentrations were measured by flame photometry (MicroNal B462) in CSF, plasma and urine samples. Chloride concentration was measured in plasma and CSF by colorimetric assay using commercial kits (Bioclin, Belo Horizonte, Brazil).

### Data and statistical analysis

Cardiovascular data was analysed in the LabChart 8.1.5 for Windows software (ADInstruments Pty Ltd, Australia). Changes in MAP and HR after drug administrations was calculated subtracting baseline levels before drug injection from the response between 6 to 11 minutes after drug injection form hexamethonium experiments or 60 minutes after drug microinjection for losartan experiments. Results are reported as mean ± standard error of the mean (SEM). Spectral analysis of the systolic interval was performed on the pulsatile blood pressure recording from rats used in experiment 1 before the hexamethonium injection. The Heart Rate Variability module of the LabChart 8.1.5 for Windows software (ADInstruments Pty Ltd, Australia) was used for the analysis and uses the Lomb Periodogram nonparametric method for spectral analysis. Time series were obtained from 4,000 to 4,500 consecutive heart beats (approximately 10 minutes of continuous recording). Spectra was divided in VLF, LF and HF bands and each was set to range from 0.0 to 0.2 Hz, 0.2 to 0.75 Hz and 0.75 to 2.50 Hz respectively. The ratio between LF and HF was used to estimate sympathovagal balance modulation. RMSSD was used to assess time-domain HR variability.

Comparisons between groups were performed either by *t*-test, one way analysis of variance (ANOVA) or two-way ANOVA for repeated measures when applicable. Bonferroni’s post-test was used for multiple comparisons following ANOVA because it allows computing confidence intervals and multiplicity adjusted *p* values. All data were statistically analysed using GraphPad Prism 6.07 software for Windows (GraphPad Software, San Diego California USA). Differences between pairs of means were considered significant when the probability of type I error was less than 5% (p < 0.05).

## Electronic supplementary material

Supplementary methods and data
