# Supplementary material for: Chronic high-sodium diet intake after weaning lead to neurogenic hypertension in adult Wistar rats
Source: Sci Rep. 2017 Jul 18;7:5655. doi: 10.1038/s41598-017-05984-9 (PMC5515999; doi:10.1038/s41598-017-05984-9)
Supplement: Supplementary file 1 — Supplementary methods and data [file 41598_2017_5984_MOESM1_ESM.pdf]

Chronic high-sodium diet intake after weaning lead to neurogenic hypertension in adult Wistar rats

[1]Paula Magalhães Gomes; [2]Renato Willian Martins Sá; [1]Giovana Lopes Aguiar; [1]Milede Hanner Saraiva Paes; [1]Andréia Carvalho Alzamora; [1]Wanderson Geraldo Lima; [1]Lisandra Brandino de Oliveira; [3]Sean D. Stocker; [2]Vagner Roberto Antunes; [1]Leonardo M. Cardoso✉

[1]Department of Biological Sciences, Institute of Exact and Biological Sciences and NUPEB, Federal University of Ouro Preto, Ouro Preto (MG), Brazil.

[2]Department of Physiology and Biophysics, Institute of Biomedical Sciences, University of Sao Paulo, Sao Paulo (SP), Brazil.

[3]Department of Medicine, Division of Renal-Electrolyte, University of Pittsburgh School of Medicine, Pittsburgh (PA), United States of America.

Supplementary Dataset

Supplementary Methods and Data

Body weight and food intake: Food intake and body weight of weaned Wistar rats were evaluated weekly up to the 12 or 15 weeks. On the week 4, 8, 12 and 15 after weaning, rats were weighed, individually housed in metabolic cages (Tecniplast SPA) for a single period of 48 hours and provided with tap water and regular or high-salt powered chow *ad libitum*. At the same period, food intake was calculated as the difference between initial and the remaining amount of powered chow 24 hours later in the metabolic cage. Thereafter, rats were again housed in collective cages and provided with free access to tap water and regular or high-salt powered chow *ad libitum.*

Supplementary Table 1S – Body weight and food intake of HS, HS Unload and respective control groups.

	Cont 12W		*HS*			*Cont 15W*		*HS Unload*		
	Mean±SEM	*n*	Mean±SEM	*n*	*p*	Mean±SEM	*n*	Mean±SEM	*n*	*p*
**Body weight (g)**										
Weaning	52±1	(16)	54±1	(16)	>0.9999	53±2	(6)	51±1	(6)	>0.9999
Week 4	204±7	(16)	210±3	(16)	>0.9999	215±5	(6)	216±8	(6)	>0.9999
Week 8	315±6	(16)	314±6	(16)	>0.9999	312±7	(6)	317±11	(6)	>0.9999
Week 12	356±6	(16)	362±5	(16)	>0.9999	368±5	(6)	368±5	(6)	>0.9999
Week 15	–		–			409±4	(6)	416±5	(6)	>0.9999
**Food intake (g/rat/24h)**										
Week 4	22±1	(10)	24±1	(11)	0.5579	23±2	(4)	24±1	(4)	>0.9999
Week 8	23±1	(10)	24±2	(11)	0.9351	23±2	(4)	23±2	(4)	>0.9999
Week 12	22±2	(10)	24±2	(11)	>0.9999	17±0.3	(4)	17±1	(4)	>0.9999
Week 15	–		–			20±1	(4)	18±0.4	(4)	>0.9999

Data were compared by two way ANOVA. Cont 12W = control for HS goup; Cont 15W = control for HS Unload group.

Supplementary Methods and Data

Water intake and urine output measurements: On the week 4, 8, 12 and 15 after weaning, rats were individually housed in metabolic cages (Tecniplast SPA) for a single period of 48 hours and provided with tap water and regular or high-salt powered chow *ad libitum*. Urine output and water intake were precisely measured gravimetrically within the last 24 hours in the metabolic cage. Thereafter, rats were again housed in collective cages and provided with free access to tap water and regular or high-salt powered chow *ad libitum.* Urine samples were centrifuged and stored at -20°C until the biochemical analysis.

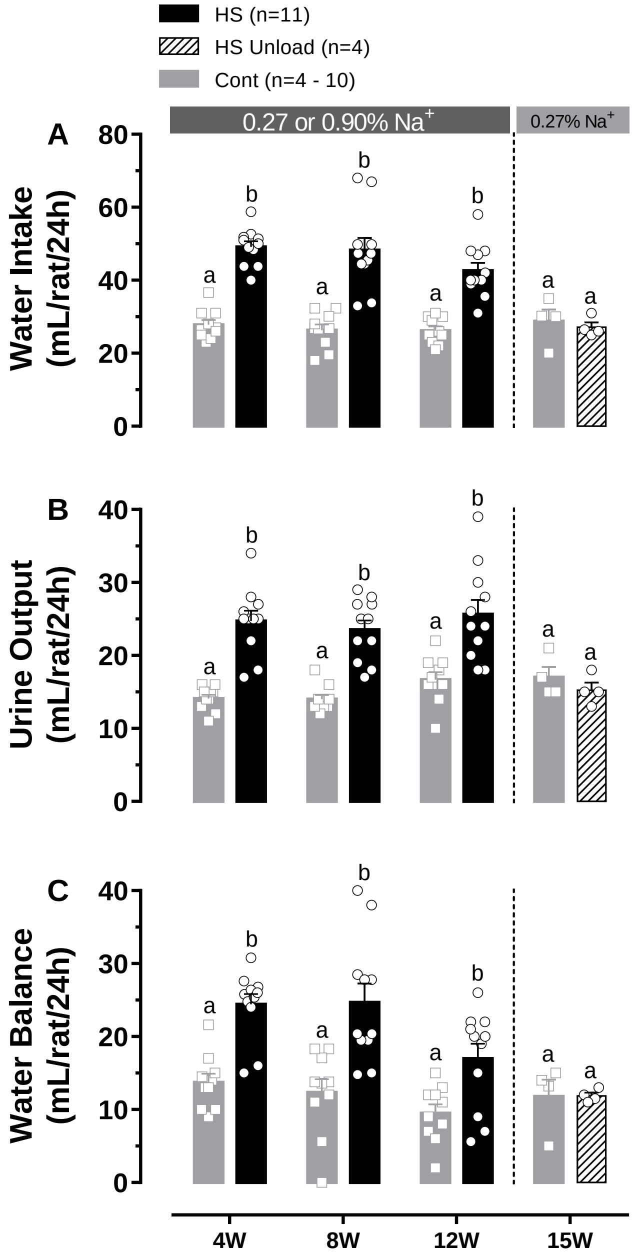

Supplementary Figure 1S – *Water intake (A), urine output (B) and water balance (C) of rats from HS, HS Unload and respective control groups*. Measurements were performed on the 4th (4W), 8th (8W), 12th (12W) and 15th (15W) weeks after weaning for 24 h in metabolic cages. Scattered squares (Cont) and circles (HS/HS Unload) represent individual values and bars represent average values with its respective SEM for each group. Differences between pairs of means are indicated by different letters. Same letter means no statistical difference. Two-way ANOVA followed by Bonferroni´s post test; $p<0.05$.

Supplementary Methods and Data

Urine samples were thawed, vortexed and a sample was taken for sodium concentration measurement using flame photometry technique (MicroNal B462). Sodium in 24 hours were calculated as the product of solute concentration by the 24 h urine output.

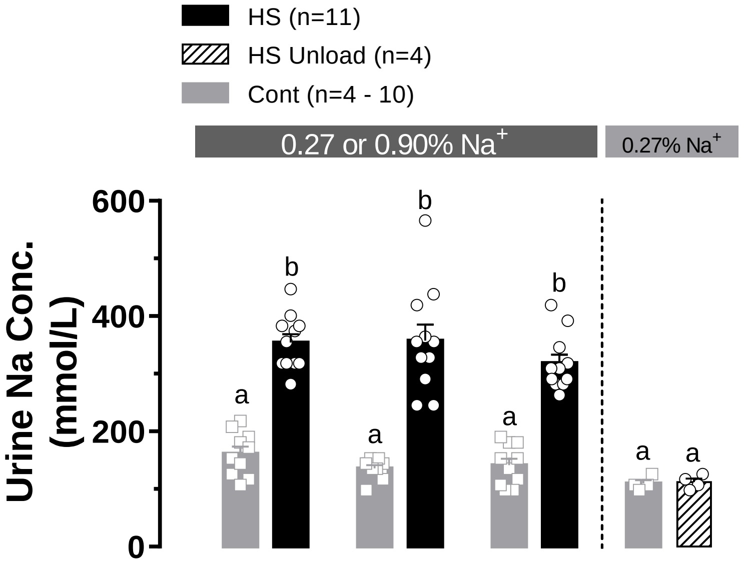

Supplementary Figure 2S – *Sodium concentration in 24-hours urine samples of rats from HS, HS Unload and respective control groups*. Measurements were performed on the 4th (4W), 8th (8W), 12th (12W) and 15th (15W) weeks after weaning for 24 h in metabolic cages. Scattered squares (Cont) and circles (HS/HS Unload) represent individual values and bars represent average values with its respective SEM for each group. Differences between pairs of means are indicated by different letters. Same letter means no statistical difference. Two-way ANOVA followed by Bonferroni´s post test; $p<0.05$.

Supplementary Methods and Data

Blood sampling for biochemical analysis: In a separate group of rats (10 HS and 10 Cont), a median incision was made in the abdomen, and abdominal cava vein was located and then injected with 20 µL of heparin (Hemofol® 5,000 IU/mL, Cristália Ltda., SP, Brazil). After thoracotomy, thoracic cava vein was sectioned right before right atrium and a 2 mL blood sample was taken. Blood samples were immediately centrifuged and plasma set apart. Osmolality in CSF and plasma samples was measured immediately after withdraw (fresh samples) The remainder was frozen in liquid nitrogen and stored at -80°C until biochemical analysis.

***Biochemical analysis*:** Creatinine and urea concentrations were measured in urine samples from the total urine volume collected over a 24-hour period. Albumin, creatinine and urea concentrations were measured in plasma samples. Colorimetric assays were performed by commercial kits (Bioclin; Quibasa Química Básica Ltda., Belo Horizonte, Brazil). Albumin determination was based on the bromocresol green method. Creatinine was measured by the modified Jaffe method (17) and urea was measured by colorimetric determination of ammonium deriving from enzymatic degradation of urea. Sodium and potassium concentrations in urine, plasma and CSF samples were measured by flame photometry (MicroNal B462). Sodium, creatinine and urea excretion in 24 hours were calculated as the product of each solute concentration by the 24 h urine output. Sodium and water balance in 24 h were calculated as the difference between intake and urinary excretion of sodium and water, respectively. Plasma and urine osmolality were measured in a freezing point micro osmometer (µOsmetteTM, Precision Systems).

Supplementary Table S2 – Biochemical test results from plasma and urine samples taken from HS rats and respective control group.

	Cont		*HS*		
**Plasma**	Mean±SEM	*n*	Mean±SEM	*n*	*p*
Albumin (mg/mL)	25.3±0.8	(10)	24.5±0.6	(10)	0.4419
Creatinine (mg/mL)	0.0030±0.0003	(10)	0.0025±0.0002	(10)	0.1288
Urea (mg/mL)	0.43±0.019	(10)	0.38±0.020	(10)	0.0546
Sodium conc. (mmol/L)	147±3.7	(10)	146±4.3	(10)	0.8891
Potassium conc. (mmol/L)	4.9±0.24	(10)	4.5±0.23	(10)	0.2351
Osmolality (mOsm/kg)	294±1.6	(10)	289±1.7	(10)	0.0949
**Urine**					
Creatinine conc. (mg/mL)	0.91±0.052	(10)	0.75±0.075	(11)	0.1097
Excr. creatinine (mg/24h)	15.1±1.31	(10)	19.1±2.60	(11)	0.2025
Urea conc. (mg/mL)	58.1±4.32	(10)	45.2±3.78*	(11)	0.0353
Excr. urea (mg/24h)	966±91.7	(10)	1,149±140.6	(11)	0.3012
Osmolality (mOsm/kg)	1,553±134	(10)	1,355±87	(8)	0.2595
**Creatinine clearance (mL/min)**	4.75±0.63	(4)	5.55±0.64	(4)	0.4087

Albumin, creatinine and urea tests were carried out using commercial kits (BioClin). Plasma sodium and potassium concentrations were measured using flame photometry. Creatinine clearance, excreted creatinine (Excr. creatinine) and excreted urea (Excr. urea) were calculated as described in the methods section. *different of control group; unpaired *t*-test. SEM = standard error mean.

Supplementary Methods

Left ventricle wall thickness/left ventricle lumen ratio calculations: To provide further insight on how the ratio was calculated, we include here the calculations we carried out to assess the data. In the histological sections, the

boundaries between the ventricle wall and lumen are often irregular. Therefore, a direct measurement of the lumen diameter and the wall thickness may result in imprecise values depending on the point where they were measured. To precisely determine ventricle lumen diameter and wall thickness, we performed a mathematical processing of the data to assess these parameters as if the wall/lumen boundaries were perfect merged circles with the same center. The following steps describe in further detail the process.

1st step: Using the software Leica Qwin Image Processing and Analysis Software (Germany), we measured the total area (lumen + left ventricle wall) of the left ventricle. The radius was then calculated using the equation 1 as if it was a perfect circle:

Equation 1:

$$R = \sqrt{\frac{A}{\pi}}$$

Where,

R = radius

A = area measured by the software.

π = 3.1415926536

2nd step: Using the software, we measured the lumen area and calculated the lumen radius as if it was a perfect circle using the equation 1.

3th step: The left ventricle wall thickness was calculated using the equation 2:

Equation 2:

$$Wt = R_{Total} - R_{Lumen}$$

Where,

Wt = left ventricle wall thickness

R_{Total} = radius of the wall + lumen circle

R_{Lumen} = radius of the lumen circle

4th Step: The left ventricle wall thickness/lumen diameter ratio was calculated using the equation 3:

Equation 3:

$$Ratio = \frac{Wt}{2 \times R_{Lumen}}$$

The ratios of the sections from each animal were averaged together and the resulting ratios of each animal was averaged to produce one mean value of ratio for each experimental group.
